# Isolation and *in vitro* selection of actinomycetes strains as potential probiotics for aquaculture

**DOI:** 10.14202/vetworld.2015.170-176

**Published:** 2015-02-13

**Authors:** Milagro García Bernal, Ángel Isidro Campa-Córdova, Pedro Enrique Saucedo, Marlen Casanova González, Ricardo Medina Marrero, José Manuel Mazón-Suástegui

**Affiliations:** 1 Department of Microbiology, Center for the Study of Bioactive Chemicals (CBQ), Central University “Marta Abreu” of Las Villas. Road to Camajuaní Km 5½. Santa Clara 54830. Villa Clara. Cuba; 2 Centro de Investigaciones Biológicas del Noroeste (CIBNOR), Instituto Politécnico Nacional No. 195, Col. Playa Palo de Santa Rita Sur, C.P. 23090. La Paz, Baja California Sur, México

**Keywords:** aquaculture, marine actinomycetes, probiotics, *Vibrio*

## Abstract

**Aim:**

This study was designed to describe a series of *in vitro* tests that may aid the discovery of probiotic strains from actinomycetes.

**Materials and Methods:**

Actinomycetes were isolated from marine sediments using four different isolation media, followed by antimicrobial activity and toxicity assessment by the agar diffusion method and the hemolysis of human blood cells, respectively. Extracellular enzymatic production was monitored by the hydrolysis of proteins, lipids and carbohydrates. Tolerance to different pH values and salt concentrations was also determined, followed by hydrophobicity analysis and genetic identification of the most promising strains.

**Results:**

Five out of 31 isolated strains showed antimicrobial activity against three *Vibrio* species. Three non-hemolytic strains (N7, RL8 and V4) among these active isolates yielded positive results in hydrophobicity tests and exhibited good growth at salt concentrations ranging from 0% to 10%, except strain RL8, which required a salt concentration >0.6%. Although these strains did not grow at pH<3, they showed different enzymatic activities. Phylogenetic analysis revealed that strains N7 and V4 have more than 99% identity with several *Streptomyces* species, whereas the closest matches to strain RL8 are *Streptomyces panacagri* and *Streptomyces flocculus*, with 98% and 98.2% similarity, respectively.

**Conclusion:**

Three actinomycetes strains showing probiotic-like properties were discovered using several *in vitro* tests that can be easily implemented in different institutions around the world.

## Introduction

Aquaculture of finfish, crustaceans and molluscs contributed to 43% of the aquatic animal food produced for human consumption in 2007 and is expected to increase further to meet the demand of the rapidly growing global population [1]. However, several factors associated with intensive farming may eventually lead to disease outbreaks caused by several pathogenic micro-organisms with increased drug resistance; such outbreaks may occur at any stage during aquatic animal development [2,3]. Members of the *Vibrio* genus are among the most frequently detected etiologic agents linked to fish, shrimp and shellfish hatcheries, which can result in high mortality rates and economic losses [4-6].

Chemical compounds, mainly antibiotics, have been extensively used to prevent and treat infectious diseases in aquatic farms. However, many of these agents can persist unaltered at the application site and surrounding environment and lead to the selection of multidrug-resistant bacteria, which can exchange their resistance determinants with animal and human pathogens through mobile genetic elements [7-10]. Therefore, the safe use and disposal of antimicrobials in aquaculture is a global public health concern that necessitates eco-friendly approaches, for example, the use of probiotic organisms that have beneficial activities.

Most probiotics proposed as biological control agents in aquaculture belong to the lactic-acid bacteria, the genus *Bacillus*, or the genera *Pseudomonas* and *Burkholderia* [11-13]. Although actinomycetes are excellent producers of antimicrobial secondary metabolites and secrete several extracellular enzymes that decompose organic matter, these microorganisms have been overlooked as protective agents in aquaculture farming. Dharmaraj noted that strains of actinobacteria belonging to the genus *Streptomyce*s might be promising probiotics in aquaculture because they produce compounds with potential bioactivity against pathogens of fish and shellfish [14,15]. However, few studies have considered actinomycetes for aquaculture purposes [16-18].

There are insufficient methods to guide the successful discovery of probiotic agents among these microbes. Here, we describe a series of *in vitro* tests that provide a broader understanding of the overall functions of these microorganisms and have the potential to accelerate the development of probiotics for aquaculture and other purposes.

## Materials and Methods

### Ethical approval

Not applicable, all experiments were conducted *in vitro*.

### Sample collection and isolation

Actinomycetes samples were collected in sterile flasks from near-shore sediments in four Central Provinces of Cuba, specifically Matanzas, Villa Clara, Cienfuegos and Ciego de Avila. The samples were immediately transported to the laboratory, stored at 4°C and processed within 72 h after collection. 1 g of each sediment sample was suspended in 9 mL of sterile seawater by vortexing, incubated for 6 min in a water bath at 55°C [19], and then serially diluted ten-fold (to 10^−5^). Aliquots (100 µL) were spread onto starch casein agar, humic acid vitamin agar, ISP2 and AMM (starch 10 g, yeast extract 4 g, peptone 2 g, agar 18 g, sea water 1 L) plates containing filter-sterilized cycloheximide (100 µg/mL) and nalidixic acid (30 µg/mL). The inoculated plates were incubated at 28°C for 28 days. The resulting colonies had different morphologies with a tough or powdery texture and a dry or folded appearance; the colonies adhered to the agar surface and had branching filaments with or without aerial mycelia, as described previously [20]. These isolated colonies (pure cultures) were picked and maintained at 4°C on ISP2 slants and at −20°C in 20% glycerol for further studies.

### Analysis of antimicrobial activity

The pathogenic strains *Vibrio alginolyticus* (CAIM 57), *Vibrio harveyi* (CAIM 1793), *Vibrio vulnificus* (CAIM 157) and *Vibrio parahaemolyticus* (ATCC 17802), obtained from the Colección de Microorganismos de Importancia Acuícola (CAIM, www.ciad.mx/caim) and the American Type Culture Collection, were selected for antagonism assay using the agar-diffusion method [21]. The actinomycetes strains were grown on starch casein agar for 7 days at 30°C. The *Vibrio* strains were inoculated on thiosulfate citrate bile salts sucrose (TCBS) agar plates for 24 h; *Vibrio* suspensions were prepared in a saline solution and the optical density (OD) at 625 nm was adjusted to 0.08-0.1. Cotton swabs from the *Vibrio* suspensions were spread on the surface of tryptone soy agar (TSA) plates supplemented with 3% sodium chloride. Subsequently, 6mm plugs were excised from the actinomycetes plates and placed on the TSA plates. The antimicrobial activity was monitored by measuring the diameter of the zone of inhibition (halos; mm) around the agar plugs after 24 h incubation at 35°C.

### Analysis of hemolytic activity

The actinomycetes strains were streaked on blood agar plates (Cat. # 211728, BD-Bioxon, Franklin Lakes, NJ, USA) containing 5% human blood and 2.5% sodium chloride (NaCl); the plates were incubated for 7 days at 30°C. Three types of hemolytic activity were examined: α (partial), ß (total) or γ (no hemolysis) [22], using the ß-hemolytic strain *V. parahaemolyticus* as control. All actinomycetes strains showing γ hemolysis patterns were used for further studies.

### Hydrophobicity analysis

Hydrophobicity was examined using the Congo red method and the Bacterial adherence to hydrocarbons (BATH) test. Selected actinomycetes strains were streaked on TSA plates containing 1% sodium chloride and 0.03% Congo red [23], and the plates were incubated at 30°C for 7 days. Strains with a reddish color were considered positive for the test, whereas strains with a translucent to white color, were considered negative. The BATH test was performed by measuring the cellular affinity for organic solvents, as described by Sweet *et al*. [24]. Briefly, the actinomycetes strains were grown in tryptic soy broth (TSB) at 30ºC for 7 days under shaking conditions. The cells were harvested by centrifugation and then washed three times with phosphate buffered saline (PBS). The OD of the cells at 540 nm was adjusted to 0.8 in PBS. Subsequently, 3 mL of each cell suspension was mixed with 1 mL of xylene and vortexed for 30 s at room temperature. After 30 min, the OD of the aqueous phase at 540 nm was measured. The percent hydrophobicity was calculated with the formula:


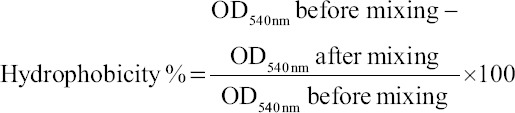


Strains were considered strongly hydrophobic when the values were >50%, moderately hydrophobic when the values were in the range 20-50%, and hydrophilic when the values were <20% [25].

### Sodium chloride and acidic pH tolerance

Starch casein agar plates containing different sodium chloride concentrations (0, 0.4, 0.6, 2, 3 and 10%) were used to analyze the sodium chloride tolerance of the actinomycetes strains [26]. Tolerance to acidic pH was examined by growing the strains in TSB at a pH of 1, 2, 3, 4 and 7.2. The isolates were seeded on agar and broth medium, incubated at 37°C for 7-15 days, and the presence or absence of growth was recorded on the 7^th^ day onwards.

### Enzymatic activity

Different substrates were used to determine the ability of the actinomycetes strains to hydrolyse macromolecular polymers such as proteins, lipids and carbohydrates. Salt was added to each culture medium at a concentration of 3% when testing enzymatic activities.

Starch hydrolysis: Amylolytic activity was determined by the radial diffusion method using marine agar plates supplemented with 2% starch [27]. Plates seeded with the test organisms (V4, RL8, N7) were incubated at 30°C for 4-5 days. Amylase production was determined by measuring the halos around the actinomycetes colonies after addition of a lugol solution to the plate surface.

Tween 80 hydrolysis: Lipolytic activity was examined by seeding the test organisms on tween-agar media [28] containing 1% Tween 80. After incubation at 30°C for 4-5 days, the tween 80-hydrolysing strains formed a precipitation halo around their colonies because of the combination of released fatty acids and Ca^2+^ ions.

Protein hydrolysis: Tryptone soy and nutrient agar supplemented with skim milk (1%) and gelatin (0.4%) [28] were used to determine the protein hydrolysis capacity of actinomycetes strains. To measure casein hydrolysis, the diameter of clear halos around colonies on skim milk plates was measured after incubation at 30°C for 4-5 days. Qualitative gelatin hydrolysis was monitored by flooding plates with a HgCl_2_ solution (HgCl_2_ 15 g, HCl 20 mL and distilled H_2_O 100 mL) after incubation at 30°C for 4-5 days. Hydrolyzed and non-hydrolyzed gelatin appeared as a clear or white opaque zone around the streaked strain, respectively.

Cellulose hydrolysis: ISP2 agar plates supplemented with 1% carboxy methyl cellulose (CMC) were used to measure the cellulolytic activity of the test organisms. Diameters of clear halos around the colonies were measured after incubation at 30°C for 4-5 days and addition of a lugol solution [17].

### DNA extraction, polymerase chain reaction (PCR) amplification and phylogenetic analysis

The genomic DNA was extracted using the PrepMan^®^ Ultra reagent (Applied Biosystems), according to the manufacturer’s instructions. The eubacterial universal primers 27f 5’AGAGTTTGATCMTGGCTCAG3’ and 1492r 5’TACGGYTACCTTGTTACGACTT3’ were used to amplify approximately 1.5 kb of the 16SrRNA gene. Each PCR mixture contained 20 µM primer, 10 µM dNTPs, 5 µl 10 × MgSO_4_ and PCR reaction buffer (Biotools, Madrid, Spain), dimethyl sulfoxide (5%), 1 µg of genomic DNA as a template, and 1U of Taq DNA polymerase (Biotools, Madrid, Spain) in a final volume of 50 µl. The PCR was performed in a master cycler thermocycler (Eppendorf) using an initial denaturation step at 94°C for 3 min, followed by 30 cycles of 94°C for 30 s, 47°C for 33 s and 72°C for 90 s and a final extension step at 72°C for 7 min. The PCR product was purified using the PureLink™ purification kit (Invitrogen). The PCR products were directly sequenced using the ABI prism dye terminator cycle sequencing kit (Perkin Elmer) and an ABI 310 Prism automated DNA sequencer (Applied Biosystems).

The sequences (> 1420 bp) were compared to those in the GenBank database using the basic local alignment search tool and to the ribosomal database project algorithm to identify known closely related sequences [29,30]. Multiple sequence alignments with reference sequences retrieved from the DDBJ/EMBL/GenBank databases were performed using MUSCLE implemented in the MEGA6 software [31], followed by manual trimming before further analysis. Phylogenetic trees were generated using the MEGA6 software with the neighbour-joining algorithm (Saitou and Nei, 1987) [32] using the Kimura 2-parameter method [33] and a bootstrapping of 1000 replicates to compute evolutionary distances and the topologies of the resultant trees, respectively. The *Escherichia coli* strain WD01 was used as the out group. The final tree was built with the Consensus program using the majority-rule consensus approach based on the neighbour-joining dataset.

## Results and Discussion

### Isolation of potential probiotic actinomycetes from marine coast sediments

Thirty-one strains characteristic of actinomycetes were isolated and differentiated from the rest of the same sample site based on their morphology, color of pigments and arrangement of mycelia. Initially, isolated colonies had a smooth appearance, but later developed aerial mycelia that either appeared floccose, granular, powdery or velvety. The color of the substrate and aerial mycelia varied from white and yellow-white to violet and pink. For a long time, it was believed that actinomycetes strains isolated from marine environments were mere runoffs of their terrestrial counterparts. Although this may be true for some isolates, this overall perception vanished after the discovery of an indigenous actinomycetes flora from marine environments [34,35]. Subsequently, several actinomycetes strains have been isolated from aquatic sediments, sponges, bryozoans and corals often as a result of efforts to find novel secondary metabolites for drug discovery [36-43]. Recent findings have shown that these microorganisms may also be part of the fish microbiota [44-46], thereby opening a door for the discovery of potential probiotics from them with probable use in fish, crustaceans and mollusk aquaculture.

### Antimicrobial and hemolytic activity of actinomycetes

**Figure-1:**
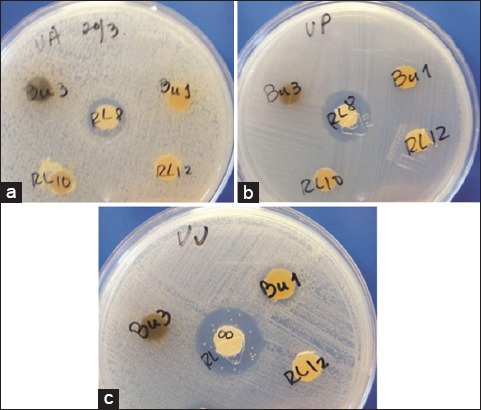
Antimicrobial activity of *Streptomyces* strain RL8 against *Vibrio harveyi* (a), *Vibrio parahaemolyticus* (b) and *Vibrio vulnificus* (c).

Five strains out of 31 actinomycetes showed antimicrobial activity against specific invertebrate pathogenic *Vibrios* (Table-1, Figure-1), representing 16.13% of all isolated strains while the strongest inhibitory effect against *V. parahaemolyticus*, *V. harveyi* and *V. vulnificus* was displayed by the strains RL8 and V4. These results are different from those obtained by You *et al*. [18], who showed that 51.1% of actinomycetes strains isolated from shrimp farms had antimicrobial activity against *Vibrios*. In contrast, our findings are in agreement with Zheng *et al*. [47], who found a similar antimicrobial effect just for 12.8% of actinobacteria tested against *Vibrio anguillarum*. None of the tested actinomycetes strains showed activity against *Vibrio alginolyticus* (Table-1). Therefore, in order to have a broad spectrum probiotic to treat vibriosis, a new actinomycetes strain having activity against this pathogen should be isolated and mixed with either strain N7, RL8 or V4.

**Table-1:** Antimicrobial and hemolytic activity of actinomycetes strains.

Strain	Inhibition halo (mm)				Haemolysis	
	
	*V. alginolyticus*	*V. parahaemolyticus*	*V. harveyi*	*V. vulnificus*	β	γ
La7	0	10	9	13	+	–
La12	0	12	8	16	+	–
N7	0	10	10	14	–	+
RL8	0	16	12	21	–	+
V4	0	18	12	22	–	+

V. alginolyticus=Vibrio alginolyticus, V. parahaemolyticus=Vibrio parahemolyticus, V. harveyi=Vibrio harveyi, V. vulnificus=Vibrio vulnificus

The hemolysis assay performed to strains exhibiting antimicrobial activity showed that only the isolates V4, RL8 and N7 were non-hemolytic (γ hemolysis), contrary to strains La7 and La12, which were hemolytic (Table-1). Toxicity tests are important not only to discard those few species of some actinomycetes genera, such as *Nocardia* and *Mycobacterium*, which have been reported as pathogenic to fish [48,49] but also those strains producing toxic secondary metabolites. Consequently, these three γ hemolytic strains are the most promising probiotic candidates from all 31 isolated actinomycetes because they are active against Vibrio and should not be either pathogenic or able of producing toxic substances that may harm fish, or shellfish. *In vivo* testing in the final host is necessary to clarify which strains are innocuous.

### Hydrophobicity test

The strainsV4, RL8 and N7showing antimicrobial activity and being non-haemolytic were also positive to the hydrophobicity test, due to their ability to bind to Congo red dye and to adsorb to xylene according to the BATH test (Table-2). Hydrophobic interactions are among the many mechanisms involved in the attachment of microorganisms to the host gastrointestinal tract [50], which means that these actinomycetes strains may interact and eventually attach to the gut, and not be easily flushed out in feces. The adhesion capacity to the gastrointestinal tract has been regarded as one of the most important criteria to select for candidate probiotic strains, thus the Congo red dye and BATH tests are useful tools for such purpose, even though *in vivo* tests will be necessary to assess the actual interaction with the host.

**Table-2:** Analysis of the hydrophobicity and salt and pH tolerance of actinomycetes strains.

Strain	Hydrophobicity test		Growth in NaCl (%)						Growth at pH				
		
	Congo red	Bath (%)	0	0.4	0.6	2	3	10	1	2	3	4	7.2
N7	+	84.6	+++	+++	+++	+++	+++	+++	–	–	–	+++	+++
RL8	+	77.6	–	–	+	+++	+++	+++	–	–	–	+++	+++
V4	+	64.4	+++	+++	+++	+++	+++	+++	–	–	–	+++	+++

### Influence of pH and salt tolerance

All actinomycetes strains had a similar behavior to different pH exhibiting no growth at pH 1-3, but growing at pH higher than 3 (Table-2). It has been estimated that the survival rate of traditional probiotics in the host’s gut is only 20-40%, being gastric acidity one of the main obstacles [51]. In spite that these actinomycetes strains cannot endure at pH between 1 and 3, higher survival rates might be expected when compared to traditional bacterial probiotics, since many actinomycetes are capable of producing spores resistant to harsh conditions such as acidity.

Given the large numbers of actinomycetes that are undoubtedly washed from the shore into the sea, it is important to distinguish between strains that have evolved in response to specific marine environmental challenges versus strains that are present as dormant spores [52]. This distinction is paramount if the strains are to be used as probiotics because they should be actively growing to exert their effect. Strains N7 and V4 grew well at any salt concentration ranging from 0 to10%, whereas strain RL8 needed more than 0.6% salt concentration in the culture media to grow. As these strains were able of growing at high salt concentrations, it is reasonable to expect that they may remain active in both marine environments and the host’s gut.

### Extracellular enzymatic activity of actinomycetes

The three most promising probiotic candidates showed different extracellular enzymatic activities (Table-3). Contrary to the strain N7, which was able of degrading all macromolecules (proteins, lipids and carbohydrates), the strain RL8 did not show lipase activity and strain V4 was unable of degrading starch and casein. Actinomycetes are notorious for their ability to produce several extracellular enzymes that decompose organic matter, such as starch, cellulose, proteins and lipids [53,54]. In aquaculture systems, these potential probiotic may help fish, shrimp and shellfish with food digestion, maximize growth, and improve water quality by degrading fecal matter and uneaten food in hatchery tanks and grow-out ponds [55]. There is probably no other microorganism in nature with such a broad spectrum antimicrobial and extracellular enzymatic activities, as those shown by actinomycetes; thus they have the potential to be ideal probiotic agents. In the present study, the strain N7 exhibited the most complete degradation capabilities of macromolecular compounds. However, the antimicrobial activity of this strain was less potent than that depicted by strains V4 and RL8. Thus, *in vivo* tests will clarify which of these three strains actually have the best probiotic effect.

**Table-3:** Enzymatic activity of selected actinomycetes strains.

Strain	Amylase	Lipase	Cellulase	Proteinase	

				Caseinase	Gelatinase
N7	+++^a^	+++	+	+	++
RL8	++++	–	+	+	+
V4	–	+++	+	–	++

a Diameter of hydrolytic halos (mm): –,0; +,< 5; ++, > 6, +++, >15

### Molecular characterization of strains

**Figure-2:**
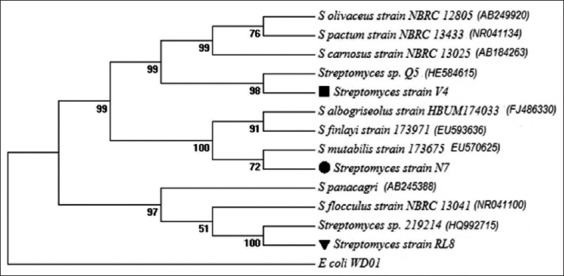
Phylogenetic tree derived from 16S rRNA gene sequences of potential probiotics and their closely related species. Numbers at nodes indicate the level (%) of bootstrap support based on neighbour-joining analysis of 1000 re-sampled datasets.

Phylogenetic analyses of 16S rRNA gene sequences revealed that the similarity of strains N7 and V4 to their closest relatives was more than 99%. Strain RL8, on the other hand, shows 98.0 and 98.2% identity with *Streptomyces panacagri* and *Streptomyces flocculus*, respectively. The 16S rDNA sequence of strains RL8, N7 and V4 were deposited in GenBank, National Center for Biotechnology Information (http://www.ncbi.nlm.nih.gov) under the accession numbers KM590924, KM590925 and KM590926, respectively. Coincidentally, the three most promising probiotic strains (N7, RL8 and V4) are members of the *Streptomyces* genera (Figure-2), which are among the richest antibiotic producing genera among the *Actinomycetales* order [56]. Similarly, it may be expected that other antibiotic-producing members, such as *Micromonospora* and *Actinoplanes*, and extracellular enzyme producing strains of actinomycetes may arise as promising candidate to probiotics in the near future.

## Conclusion

Because of their ability to produce antimicrobial secondary metabolites and extracellular enzymes that decompose organic macromolecules, some actinomycetes strains have a great probiotic potential. The identification of these actinomycetes strains with potential probiotic activity should begin with the isolation of strains from healthy animals or their surrounding environment or both. Subsequently, their antimicrobial activity and toxicity must be examined using a hemolytic assay. Other properties, to be considered, are tolerance to salt and different pH values as well as the *in vitro* ability to colonize the host gastrointestinal tract. Finally, the ability to degrade several macromolecular substrates should be examined. The ideal actinomycetes probiotic strain should have the following properties: No toxicity, broad-spectrum antimicrobial activity against pathogenic microbes, activity in the aquatic environment, high survival rate in the animal gut, decomposition of the widest possible spectrum of macromolecular substrates in the host and the environment, and a synergistic effect when used in a probiotic mixture. The actinomycetes strains V4, RL8 and N7 are strong candidates to have most of these beneficial traits. We propose that this protocol can be used to identify some actinomycetes strains that can be effective probiotics for fish and shellfish aquaculture.

## Authors’ Contributions

MGB, RMM and JMMS designed the study. MGB, RMM and MCG performed the experiments. AICC, PES and JMMS gave technical guidance during the experiment. MGB, RMM and JMMS analyzed the data. MGB, RMM, AICC, PES and JMMS drafted and revised the manuscript. All authors read and approved the final manuscript.
